# Occupational Hantavirus Infections in Agricultural and Forestry Workers: A Systematic Review and Metanalysis

**DOI:** 10.3390/v13112150

**Published:** 2021-10-25

**Authors:** Matteo Riccò, Simona Peruzzi, Silvia Ranzieri, Nicola Magnavita

**Affiliations:** 1 AUSL–IRCCS di Reggio Emilia, Servizio di Prevenzione e Sicurezza Negli Ambienti di Lavoro (SPSAL), Local Health Unit of Reggio Emilia, Via Amendola n.2, I-42122 Reggio Emilia, Italy; 2 AUSL–IRCCS di Reggio Emilia, Laboratorio Analisi Chimico Cliniche e Microbiologiche, Ospedale Civile di Guastalla, I-42016 Guastalla, Italy; simona.peruzzi@ausl.re.it; 3 School of Occupational Medicine, Department of Medicine and Surgery, University of Parma, Via Gramsci n.14, I-43123 Parma, Italy; silvia.ranzieri@unipr.it; 4 Postgraduate School of Occupational Medicine, Università Cattolica del Sacro Cuore, I-00168 Rome, Italy; nicola.magnavita@unicatt.it; 5 Department of Woman/Child & Public Health, Fondazione Policlinico Universitario Agostino Gemelli IRCCS, I-00168 Rome, Italy

**Keywords:** hantaviruses, work-related disease, climate change, public health, sectors of activity, workers, zoonoses

## Abstract

Hantaviruses are zoonotic pathogens that can cause serious human disorders, including hemorrhagic fever with renal syndrome and hantavirus cardiopulmonary syndrome. As the main risk factor for human infections is the interaction with rodents, occupational groups such as farmers and forestry workers are reportedly at high risk, but no summary evidence has been collected to date. Therefore, we searched two different databases (PubMed and EMBASE), focusing on studies reporting the prevalence of hantaviruses in farmers and forestry workers. Data were extracted using a standardized assessment form, and results of such analyses were systematically reported, summarized and compared. We identified a total of 42 articles, including a total of 28 estimates on farmers, and 22 on forestry workers, with a total workforce of 15,043 cases (821 positive cases, 5.5%). A pooled seroprevalence of 3.7% (95% confidence interval [95% CI] 2.2–6.2) was identified in farmers, compared to 3.8% (95% CI 2.6–5.7) in forestry workers. Compared to the reference population, an increased occurrence was reported for both occupational groups (odds ratio [OR] 1.875, 95% CI 1.438–2.445 and OR 2.892, 95% CI 2.079–4.023 for farmers and forestry workers, respectively). In summary, our analyses stress the actual occurrence of hantaviruses in selected occupational groups. Improved understanding of appropriate preventive measures, as well as further studies on hantavirus infection rates in reservoir host species (rodents, shrews, and bats) and virus transmission to humans, is needed to prevent future outbreaks.

## 1. Introduction

Hantaviruses (family *Hantaviridae*) are monopartite, trisegmented, negative-stranded enveloped RNA viruses belonging to the order of *Bunyavirales* [1,2,3,4]. Usually carried by rodents and insectivores [3], but also chiropters, and even reptiles and fish [5], hantaviruses have been recognized worldwide and are heterogenous, mirroring the evolutive history of their hosts [1]. According to their geographical distribution and to the clinical features of human infections, hantaviruses are often dichotomized in Old World/Eurasian and New World/American species [1,6,7]. New World hantaviruses (e.g., Andes virus, ANDV, the Sin Nombre virus, and SNV) usually cause a severe syndrome characterized by pneumonia and cardiopulmonary dysfunction (i.e., hantavirus cardiopulmonary syndrome or HCPS), whose case fatality rate may reach 40%. Old World hantaviruses are responsible for the large majority of notified cases; most of them occur in Mainland China as a syndrome characterized by renal failure and hemorrhagic manifestations (hemorrhagic fever with renal syndrome or HFRS), with an average annual incidence of 0.83/100,000 inhabitants and a case fatality rate up to 15% [6,8]. According to the European Centre for Disease Prevention and Control (ECDC) [9,10], in the last decade the annual incidence rate ranged from 0.4 to 1.1 cases/100,000 persons for most of Central and Eastern European countries, with a total disease burden ranging between 9000 to 15,000 cases/year, mostly associated with Puumala virus (PUUV) and Dobrava–Belgrade virus (DOBV) infections [2,9,10]. However, clinical features of hantavirus infections suggest that such figures may be largely underestimated. While DOBV infections may evolve in HFRS, PUUV usually elicits a milder syndrome, i.e., nephropathia epidemica (NE), which is generally not associated with major hemorrhagic symptoms, has an extremely low case fatality rate (around 0.4%), and may also go undiagnosed [8]. Moreover, serological studies suggest that symptomatic cases represent only a small fraction of the actual burden of disease, as the majority of human infections occur unnoticed, either asymptomatic or as a mild flu-like syndrome characterized by high fever, malaise, and myalgia [1,2].

Human hantavirus infection is usually classified as a direct zoonosis (i.e., orthozoonosis) [11], as recipients become directly infected through inhalation of aerosols, including excreta of the hosts (i.e., urine, feces, saliva), or more rarely by their bites [1,2,3]. Even though inter-human spreading has been reported for some strains of the Andes virus [1], and HFRS has been occasionally acquired by means of blood transfusions [4], the main risk factor for hantavirus infection is represented by occupational, domestic and/or recreational activities that favor human–rodent contact, mainly including forestry workers, agricultural workers, and military personnel [1]. Therefore, the present systematic review and meta-analysis was undertaken to summarize available evidence about the risk of hantavirus infections among agricultural and forestry workers in order to ascertain the hantavirus seroprevalence in the aforementioned occupational groups. Such a review can result in prevention strategies to specifically characterize high-risk groups and then minimize the occurrence of occupational or work-related hantavirus infections.

## 2. Materials and Methods

This systematic review has been conducted following the PRISMA (prepared items for systematic reviews and meta-analysis) guidelines [12,13]. We searched two scientific databases (i.e., PubMed and EMBASE) for relevant studies until 30/06/2020, without any chronological restriction. The search strategy was a combination of the following keywords (free text and Medical Subject Heading [MeSH] terms): (“Hantavirus disease*” OR “Hantavirus Cardiopulmonary Syndrome” OR “HCPS” OR “Hemorrhagic Fever with Renal Syndrome” OR “HFRS” OR “Nephropathia epidemica”) AND («occupation*» OR «work-related») AND («epidemiology» OR «prevalence» OR «frequency» OR «occurrence»). Records were handled using a references management software (Mendeley Desktop Version 1.19.5, Mendeley Ltd. 2019), and duplicates were removed.

Documents eligible for review were original research publications available online or through inter-library loan. Articles were required to be written in Italian, English, German, French or Spanish, the languages spoken by the investigators. Studies included were national and international reports, case studies, cohort studies, case–control studies and cross-sectional studies. Only articles reporting on agricultural settings and/or forestry workers were retrieved. Retrieved documents were excluded if: (1) full text was not available; (2) articles were written in a language not understood by reviewers; (3) reports lacked significant timeframe (i.e., the prevalence year); (4) a proper definition of the occupational settings was lacking; (5) reports lacked definition of the geographical settings, or it was only vaguely defined.

Two independent reviewers reviewed titles, abstracts, and articles. Titles were screened for relevance to the subject. All articles reporting original studies, not meeting one or more of the exclusion criteria, were retained for full-text review. The investigators independently read full-text versions of eligible articles. Disagreements were resolved by consensus between the two reviewers; where they did not reach consensus, input from a third investigator (MR) was obtained. Further studies were retrieved from reference lists of relevant articles and consultation with experts in the field.

Data abstracted included: (a) setting of the study: prevalence year, country; (b) occupational setting of the sampled cases (i.e., either agricultural or forestry workers); (c) total number of prevalent cases; (d) number of reference population; (e) characteristics of the pathogen (if available, i.e., Old World hantaviruses vs. New World hantaviruses).

We first performed a descriptive analysis to report the characteristics of the included studies. Crude prevalence figures were initially calculated: if a study did not include raw data, either as number of prevalent cases, or referent population, such figures were reverse-calculated from available data. In cases of studies dealing with the same population in various points of time, estimates were calculated for the more recent study by removing cases previously included in earlier reports.

Pooled prevalence estimates were then calculated by means of prevalent cases per 100 population. To cope with the presumptive heterogeneity in study design, we opted for the random effect model. The amount of inconsistency between included studies was estimated by means of I^2^ statistic (i.e., the percentage of total variation across studies that is due to heterogeneity rather than chance). In the present paper, I^2^ values were categorized as follows: 0 to 25% low heterogeneity; 26% to 50% moderate heterogeneity; ≥ 50% substantial heterogeneity. To investigate publication bias, contour-enhanced funnel plots representing Egger test for quantitative publication bias analysis (at a 5% of significance level) were generated. In case of asymmetry at the funnel plots, outliers were excluded irrespective of the results of Egger’s test. In fact, Egger’s test may yield false positive results if fewer than 10 studies were included. Radial plots were then calculated and visually inspected to rule out small study bias.

All calculations were performed in R (version 4.0.3) [14], and RStudio (version 1.4.1717; RStudio, PBC; Boston, USA) software by means of the meta package (version 4.9-9). The meta package is an open-source add-on for conducting meta-analyses.

## 3. Results

Initially, 257 entries were identified, including a total of 144 abstracts from PubMed, and 113 from EMBASE: as 150 of them were duplicated across the sources, 107 entries were initially screened.

**Figure 1:**
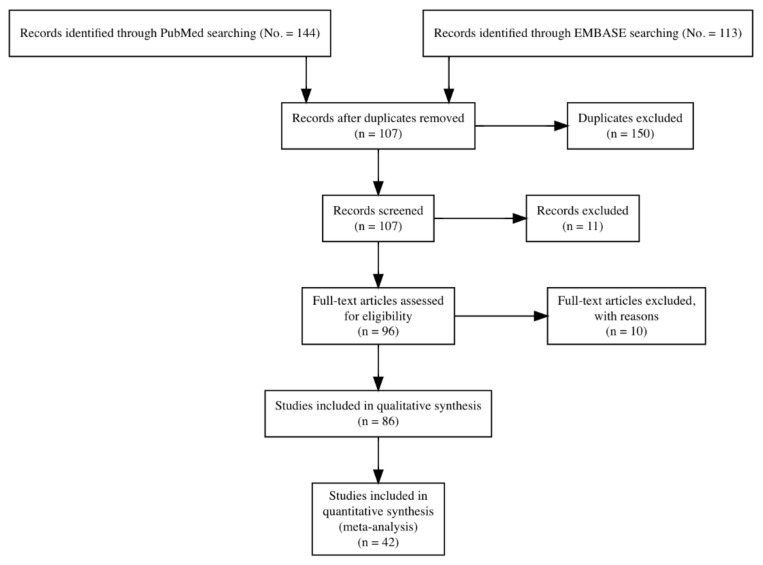
PRISMA flow chart for retrieved studies.

After applying the inclusion and exclusion criteria (Figure 1), a total of 42 articles were included in the analyses and summarized, with a total of 28 estimates on agricultural workers and 22 on forestry workers, from 20 studies reporting on agricultural workers [15,16,17,18,19,20,21,22,23,24,25,26,27,28,29,30,31,32,33,34,35,36,37,38], 14 on forestry workers [39,40,41,42,43,44,45,46,47,48], and eight further studies reporting on both occupational groups [15,18,21,22,23,49]. In one of the earlier studies, authors reported agricultural and forestry workers as a single exposure group, and the estimates were therefore included in both sub-analyses [30].

All retrieved studies are summarized in Table 1.

Briefly, a total workforce of 15,043 individuals was involved in the analyses, with 821 positive cases (5.4%). As summarized in Table 2, most of estimates were from the Old World, with 27 studies from Europe (64.3%), followed by the New World (i.e., 21.4%; of which, 7.1% for North America, and 14.3% for South and Central America), Asia (9.5%), Africa (4.8%), with a similar representation of the sampled working populations. Around a third of the studies (35.7%) were performed up to 2000, with 12 (28.6%) reporting from the following decade, and 15 from the decade 2011–2020 (35.7%).

### 3.1. Studies on Agricultural Workers

**Figure 2:**
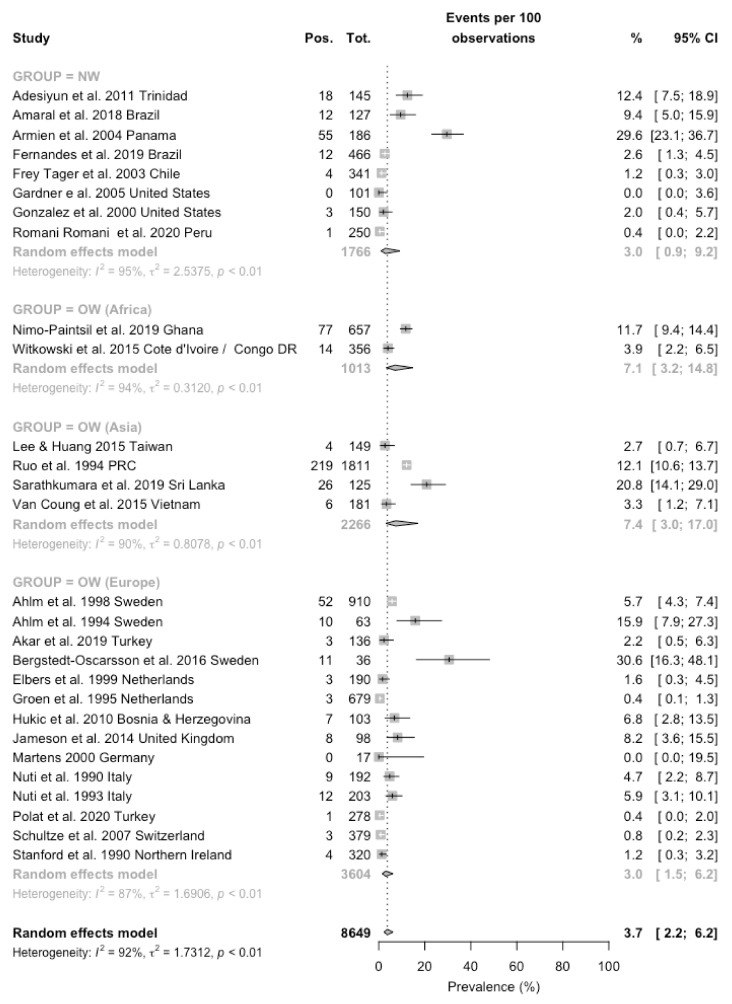
Forest plot representing the estimated pooled prevalence for seropositive status for hantaviruses among agricultural workers. Pooled prevalence rate was estimated in 3.7% (95% CI 2.2–6.2), with estimates that were considerably greater in studies performed in Asian countries (7.4%, 95% CI 3.0–17.0), followed by African countries (7.1%, 95% CI 3.2–14.8), European (3.0%, 95% CI 1.5–6.2) and American countries (3.0%, 95% CI 0.9–9.2). Notes: OW = Old World (i.e., Eurasia and Africa); NW = New World (North and Central/South America).

Prevalence of the seropositive status ranged from zero cases [51,55], to 30.6% in a more recent survey from Sweden [31]. When the prevalence rates were broken down by geographic areas, they ranged from 3.0% (0.9–9.2) in eight estimates from the New World [16,17,18,26,27,34,51,52], 3.0% (1.5–6.2) in 14 estimates from European countries [15,21,22,23,24,25,30,31,35,36,49,50,53,55], to 7.1% (3.2–14.8) in two studies from Western Africa [19,20], and eventually 7.4% (3.0–17.0) in four estimates from Asian Countries [28,29,33]. When estimates for New World were dichotomized for North vs. Central and South America, seroprevalence rates were 1.2% (0.4–3.6) and 4.5% (1.3–14.1), respectively (data not shown in Figure 2). A pooled prevalence was then estimated in 3.7% (2.2–6.2), with a substantial heterogeneity (I^2^ = 91.9%, Q = 334.02, τ^2^ = 1.713, *p* < 0.001) (Figure 2).

Assuming the occurrence of the seropositive status in European studies as a reference (Table 3), all other geographic areas exhibited an increased rate, with a rate ratio (RR) equal to 1.701, 95% CI 1.321–2.189, for studies from North and South America, RR 2.525, 95% CI 1.945–3.276 for African studies, and RR 3.219, 95% CI 2.617–3.959 for Asian-based estimates. When the estimates for North and Central/South America were calculated individually, an increased risk was associated only with Central and South America (RR 1.926, 95% CI 1.494–2.483 vs. RR 0.342, 95% CI 0.110–1.067 for North America). On the contrary, no significant differences in the seroprevalence status were identified in studies performed in the decades 2001–2010 (RR 0.730, 95% CI 0.597–0.892) [15,18,26,49,51], and 2011–2020 (RR 0.935, 95% CI 0.768–1.138) [16,17,19,20,27,29,33,34,36,38,53] compared to earlier reports [21,22,23,24,25,28,30,50,51,55].

**Figure 3:**
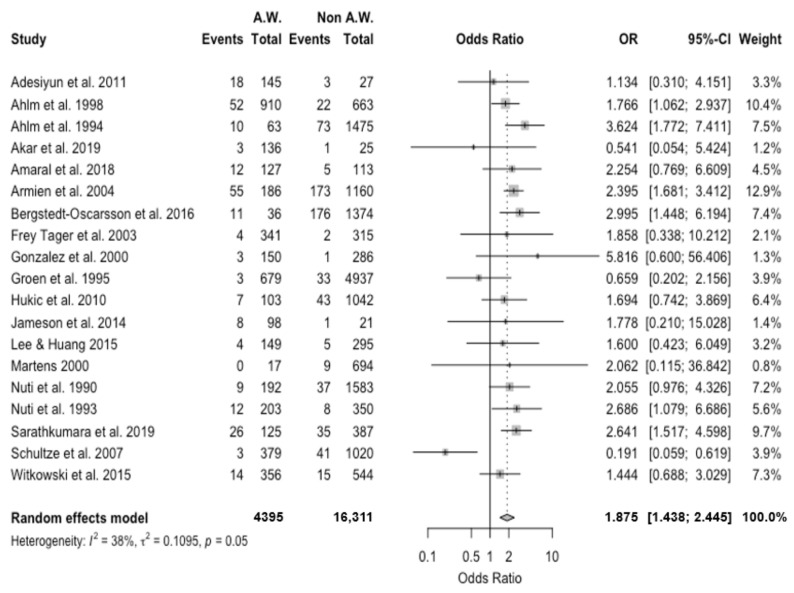
Forest plot representing the association of positive status for hantavirus serology (i.e., “Event”) in Agricultural Workers (AW) compared to the reference population (Non AW). In summary, seropositivity for Hantavirus was associated with the occupational status as AW with an odds ratio (OR) equal to 1.875, 95% confidence interval (95% CI) 1.438–2.445.

Occurrence of the seropositive status was compared with the reference non-exposed population when available [15,16,18,19,21,22,23,26,29,30,31,33,34,49,50,52,55,61]. A pooled OR equals to 1.875, 95% CI 1.438–2.445, was eventually calculated, with moderate heterogeneity (I^2^ = 37.9%, τ^2^ = 0.109, Q = 28.97, *p* = 0.048) (Figure 3).

### 3.2. Studies on Forestry Workers

**Figure 4:**
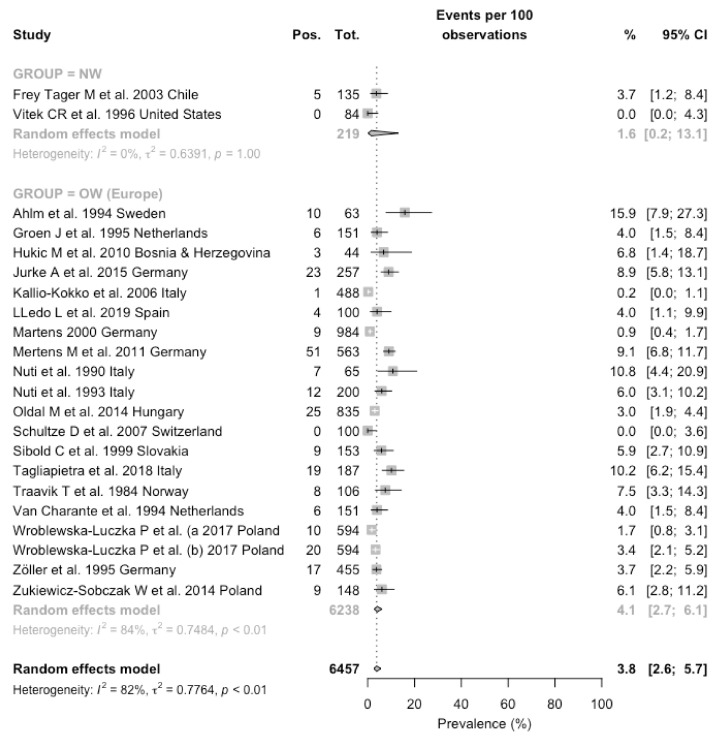
Forest plot representing the estimated pooled prevalence for seropositive status for hantaviruses among forestry workers. Pooled prevalence rate was estimated in 3.8% (95% CI 2.6–5.7), with estimates that were considerably greater in studies performed in European countries (4.1%, 95% CI 2.7–6.1), compared to North and South American countries (1.6%, 95% CI 0.2–13.1). Notes: OW = Old World (i.e., Eurasia and Africa); NW = New World (North and Central/South America).

Estimates on forestry workers were available only from European [15,21,22,23,30,40,41,42,43,44,45,46,47,49,55,57,58,59,60] and American regions [18,48]. Two of the European reports [45,59] seemly reported on the same occupational groups (i.e., forestry workers from Poland), focusing either on PUUV [45] or DOBV [59] but no specific disclosure was provided by study authors. Prevalence of the seropositive status ranged from zero cases [48] in a survey from North America (Southwestern USA) and in the report from Schultze et al. from Switzerland [49], to 15.9% in a study from Sweden [30] that included farmers and forestry workers in the same exposure groups. When the prevalence rates were broken down by geographic areas, they ranged from 1.6% (0.2–13.1) in the estimates from New World, to 4.1% (2.7–6.1) in the 20 estimates from European countries. A pooled prevalence was then estimated in 3.8% (2.6–5.7), with a substantial heterogeneity (I^2^ = 82.4%, Q = 119.05, τ^2^ = 0.776, *p* < 0.001) (Figure 4).

However, a comparison between prevalence rates that assumed European studies as the reference ones identified a correspondent RR 0.572, 95% CI 0.238–1.375 for reports from North and South America. Moreover, when estimates for North and Central/South America were calculated individually, no increased risk was eventually identified (RR 0.928, 95 CI 0.389–2.212 for North America; 0.149, 95% CI 0.001–2.371 for South America). Similarly, no significant differences in the prevalence status were identified in studies performed in the decades 2001–2010 (RR 1.295, 95% CI 0.936–1.792) [15,18,42,49], and 2011–2020 (RR 1.163, 95% CI 0.880–1.537) [39,40,41,43,44,45,47,59] when compared to previous reports [21,22,23,30,48,55,56,57,58,60].

**Figure 5:**
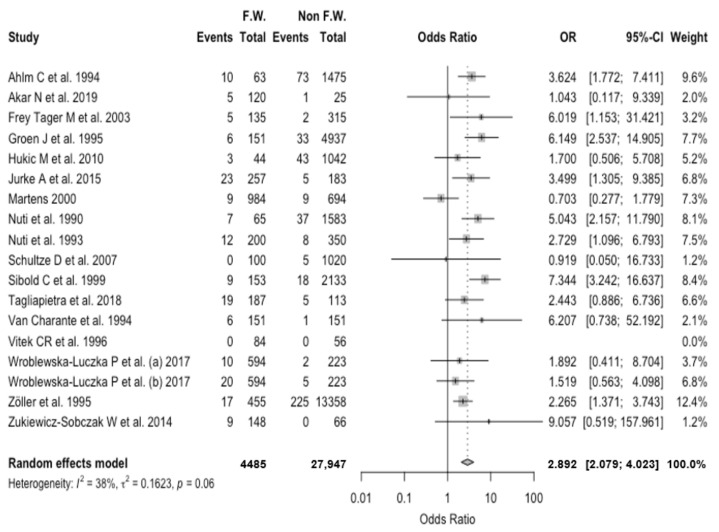
Forest plot representing the association of positive status for hantavirus serology (i.e., “Event”) in forestry workers (FW) compared to the reference population (non FW). In summary, seropositivity for hantavirus was associated with the occupational status as AW with an odds ratio (OR) equal to 2.892, 95% confidence interval (95% CI) 2.079–4.023.

As shown in Figure 5, a pooled OR equal to 2.892, 95% CI 2.079–4.023 was eventually calculated, with moderate heterogeneity (I^2^ = 38.1%, τ^2^ = 0.162, Q = 25.84, *p* = 0.056).

### 3.3. Comparison between Agricultural and Forestry Workers

**Figure 6:**
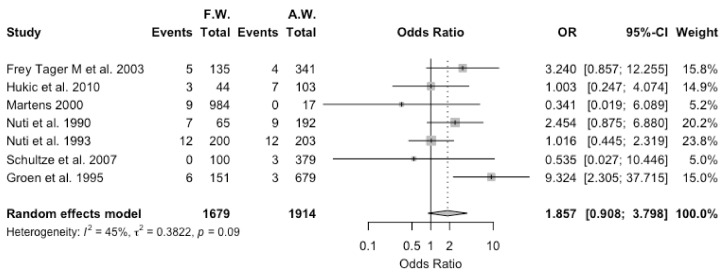
Forest plot comparing the positive status for hantavirus serology (i.e., “Event”) in forestry workers (FW) and agricultural workers (AW) in studies that reported on both occupational groups. In summary, working as FW was associated with seropositive status with an odds ratio (OR) equal to 1.857, 95% confidence interval (95% CI) 0.908–3.798.

Estimates for agricultural workers and forestry workers were compared for the eight studies that reported on both occupational groups. However, as in one of the studies [30] agricultural and forestry workers were included in the same exposure group, it was excluded from the final calculations. The seven studies [15,18,21,22,23,49,55] included a total of 1679 forestry and 1914 agricultural workers, with 42 (2.5%) and 38 (2.0%) seropositive workers. A pooled OR of 1.857, 95% CI 0.908–3.798 was eventually estimated, with moderate heterogeneity (I^2^ = 44.7%, τ^2^ = 0.382, Q = 10.85, *p* = 0.093) (Figure 6). In other words, no significant differences between agricultural and forestry workers were found for studies that included both occupational groups.

### 3.4. Publication Bias

**Figure 7:**
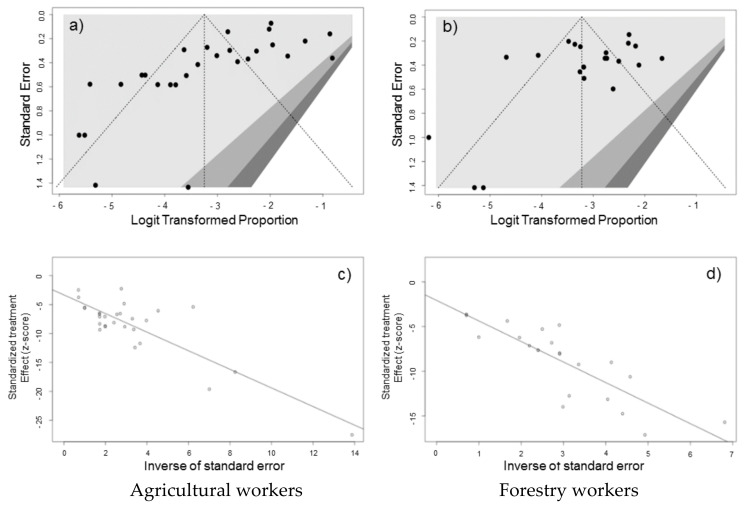
Border-enhanced funnel plots for studies included in the meta-analysis for agricultural workers (**a**) and forestry workers (**b**). Visual inspection of contour-enhanced funnel plots suggested substantial evidence of publication bias for both subgroups, but this was substantially rejected by Egger test for forest workers (i.e., t = −1.81, df = 20, *p*-value = 0.0857) and confirmed for agricultural workers (t = −3.92, df = 26, *p*-value = 0.0006 for forestry workers). On the other hand, in radial plots, the studies on agricultural workers (**c**) and forestry workers (**d**) were substantially scattered across the regression line, suggesting no significant small study effect.

The presence of publication bias was evaluated using funnel plots and regression tests for funnel plot asymmetry, separately for studies reporting on agricultural and forestry workers. Each point in funnel plots represents a separate study and asymmetrical distribution indicates the presence of publication bias. First, studies’ effect sizes were plotted against their standard errors and the visual evaluation of the funnel plot suggested a significant publication bias (Figure 7a,b). Such subjective evidence from the funnel plot was only partially confirmed after the regression test. In fact, Egger test ruled out publication bias for forest workers (i.e., t = −1.81, df = 20, *p*-value = 0.0857) while it was confirmed for agricultural workers (t = −3.92, df = 26, *p*-value = 0.0006 for forestry workers). On the other hand, in radial plots for studies on agricultural workers and forestry workers (Figure 7c,d), estimates were substantially scattered across the regression line, suggesting no significant small study effect.

## 4. Discussion

During the last decades, hantaviruses have emerged as endemic and often ignored pathogens in most of Western Europe [50,62,63,64,65,66], but also in North and South America [51,52,67,68,69,70,71,72,73]. Our meta-analysis on hantavirus in agricultural and forestry workers estimated a pooled seroprevalence of 3.7% and 3.8%, respectively, with substantial heterogeneity across the assessed areas, but highly consistent across the assessed timeframe (i.e., 1972–2020). Available estimates not only often exceeded those of the general population from the same countries (e.g., <1% in Switzerland, 1.7% in Slovenia, to 1–2% in Austria, 1–3% in Germany) [49,66,74,75], but suggested that the pathogens do circulate even in countries where no official notification of hantavirus infections has been reported to date (e.g., Italy) [76].

An increased occurrence of the seropositive status was identified for both occupational groups (i.e., OR 1.857, 95% CI 0.908–3.798 and OR 2.892, 95% CI 2.079–4.023 in agricultural and forestry workers, respectively) when compared to the reference healthy population, with no significant differences in-between (OR 1.857, 95% CI 0.908–3.798). Again, such results were not unexpected: for example, a recent meta-analysis on the seroprevalence of hantavirus infections in Italy identified an increased risk of seropositivity for all occupational groups that favor human–rodent interaction, including farmers (OR 3.053, 95% CI 1.787 to 5.103), rangers (OR 2.788, 95% CI 1.047, 7.488), and more generally speaking, the forestry workers as a whole (OR 2.343, 95% CI 1.519 to 3.599) [76].

Furthermore, the significant heterogeneity of the retrieved studies, with estimates that in some areas were greater than 10% [20,21,26,28,29,30,31,34,43], was consistent with available evidence, and substantially points towards two main risk factors, i.e., socioeconomic development of the targeted population, and the ecology of the rodent hosts [4,6,54,74,77,78], that in turn are a direct consequence of the biology of hantaviruses.

Hantaviruses are spread to the environment through the competent host’s urine, feces or saliva [79], with the subsequent transmission to the human hosts through inhalation of aerosols laden with viral particles. As hantaviruses may remain infective up to 15 days in a temperate environment, and up to 24 h for environmental temperature up to 37 °C [79,80], a direct and known interaction with the competent hosts is not required and may occur unnoticed. Therefore, living in a rural environment and/or in precarious, non-hygienic settings, and any interaction with environments potentially shared by the competent hosts represent the most significant risk factors for hantavirus infection [6,50,81,82]. In other words, any variation and/or combination of the aforementioned factors directly influences the actual risk profile of the targeted population.

For instance, the occupational groups we studied are at high risk of interacting with rodent hosts, whose ecology is in turn highly variable, not only at geographical level, but also over time, because of a complicated interaction with their environment [1,83,84]. For example, a German study in 1995 estimated a seroprevalence ranging between 1 and 2% of the general population, but 10 years later the prevalence rates climbed to 7% in the epidemic areas of Baden-Württemberg and Lower Bavaria [39,60,63], with a notification rate that slowed down in the following decade [9,65]. At the same time, a seasonal pattern emerged that is presumptively driven by food supplies. Warmer and humid winter, associated with intrinsic effect of viral infection, eventually result in early reproduction and population irruption in the following year [1,9,65,83,84], with higher rates in humans during spring. Even though climate change has guaranteed an appropriate setting for an increased spreading of hantavirus to the high-risk groups, our study identified no significant differences in prevalence rates, but several explanations are possible. First, most of the studies lacked an appropriate follow-up. In fact, among the studies we were able to retrieve, only two estimates focused on the same geographic area (i.e., the Autonomous Province of Trento, Northeastern Italy) [42,43], and the prevalence rates skyrocketed from 0.2% in 2006, to 10.2% in 2018. Second, most of the studies that were published during the last decade were performed in areas where previous estimates were not available, such as Eastern Europe (e.g., Bosnia [15], Hungary [41], Poland [44,45,59,85]), Turkey [35,36], South-East Asia (Taiwan [33], Vietnam [38]), Western Africa [19,20], and rural areas of Brazil [16,17]. Third, it should be kept in mind that hantaviruses are only limitedly cross-reactive: while modern technologies have considerably improved our diagnostic options, a critical appraisal of available studies cannot rule out that some diagnoses may have been lost because of the high specificity of the diagnostic assays. For example, studies from North America have focused on the Sin Nombre virus [48,52,67] that is by far the most important pathogenic hantavirus in North America because of its high case-fatality ratio and identified a seroprevalence rate of three cases out of 335 workers (i.e., 0.9%). Notwithstanding the very high risk of human–rodent interaction because of the socio-economic characteristics of some occupational groups [51,52], no data on other hantavirus pathogens were provided. Similarly, some recent reports from Poland have reported on PUUV and DOBV, separately [45,59], even though the characteristics of the study population hint towards its substantial overlapping, and no information on other pathogens (e.g., SEOV) or cross-seropositivity was provided. In other words, the actual seroprevalence rate among this subset of forestry workers may have been largely underestimated.

Even though pooled estimates hint towards an increased risk for seropositivity in the targeted occupational groups compared to the general population that in forestry workers peaked up to 200%, we cannot rule out that even such figures may have underestimated the actual occupational risk. In fact, most of the “reference” population included in the analyses were drawn from the same communities of the occupational groups [16,17,30,31,50], or from the parent companies, being classified as “non-exposed” by means of an arbitrary cut-off in the time spent in outdoor tasks [18,40,44,45,59], or through the analysis of specifically designed questionnaires [17,18,19,20]. Even though some larger studies [49,60] included as a reference group “healthy” subjects drawn from the general population, the design usually lacked an appropriate appraisal of individual risk factors.

Moreover, the same working definition of farmers and farm workers across the various studies was inconsistent. While most of the European-based researched reported on subjects that usually owned their field [25,30,37,50,61], North-American research extensively included a migrant workforce [51,52], while Asian, South-American and African papers mostly included subjects from a low-socioeconomic status, that were at higher risk for direct and indirect interaction with rodents and their excreta at peridomestic level [16,17,19,20,26,29,38].

### Limits

Despite the potential interest, our study is affected by several limitations. Firstly, it shares the implicit limits of all meta-analyses, being highly dependent on the quality of the original studies [86,87], and potentially affected by their high heterogeneity [87]. Unfortunately, not only was the quality of the studies we were able to retrieve highly heterogenous, but most of them were affected by significant shortcomings that ranged from the same definition of occupational groups, to a large timeframe in the sampling collection. As pointed out by Rou et al. [28], seropositivity among high-risk groups may increase rapidly, meaning that studies performed over a larger timeframe may be scarcely comparable to those completed in a shorter timeframe. For example, Groen et al. reported on a 12-year timeframe (1972–1994) [23], compared to the 5 years in Martens and Nuti [21,22,55], and the 4 years from Kallio-Kokko et al. [42].

Likewise, the comparison of seroprevalence rates across various studies and different decades is complicated by the various methodologies of laboratory assessment. For instance, the most frequently reported laboratory assays, i.e., enzyme immunoassay (EIA) and its subsequent iteration as enzyme-linked immunosorbent assay (ELISA) and immunofluorescent assay (IFA) are quite reliable, rapid and not very expensive techniques, that share the basic blueprint represented by the antigen-antibody reaction, where the antibodies are tagged with fluorescent dye (IFA), or enzymes color either directly or indirectly the antigen–antibody reaction (EIA, ELISA) that then can be read with the naked eye or with a spectrophotometer. Unfortunately, such assays are less sensitive than Western blotting (WB): in WB, a synthetic or animal-derived antibody (i.e., the primary antibody) that recognizes and binds to a specific target protein is added to an electrophoresis membrane containing the target protein. A secondary antibody is added, which recognizes and binds to the constant region of the primary antibody. The secondary antibody is visualized through various methods (e.g., staining, immunofluorescence, and radioactivity) allowing indirect detection of the specific target protein. Because of its greater sensitivity, WB may give positive results even if other serological tests are negative. Unfortunately, as performing WB is far more expensive with increased laboratory turnaround time than EIA/ELISA/IFA, certain studies have reserved this more accurate approach as a confirmatory test [35,36,41,49], and such factors may have significantly contributed to the high heterogeneity of the pooled estimates [23,39,60,83,88]. Consequently, not only may the comparison of available estimates be even more problematical, but most of reported estimates may have significantly underestimated the actual seroprevalence among sampled groups. Not coincidentally, while the study of Schultze identified an ELISA-based prevalence of 9.4%, that in turn dropped to 0.3 to 0.5% in immunofluorescence and/or immunoblot assays. Similarly, a study on the blood donors from St. Gallen Switzerland found a prevalence of 3.8% at median fluorescence intensity, that dropped to 0.6% in IFA [49,75].

## 5. Conclusions

In summary, collected seroprevalence studies collectively confirm that occupational and/or work-related hantavirus infections globally occur, at least in farmers and/or forestry workers from areas characterized by the likely interaction between humans and rodents. Because of the characteristics of the studies, we were able to retrieve, we cannot rule out that the occurrence of human infections may be extensively underestimated. As hantavirus may be a significant cause of acute and chronic disease, our data not only suggest that occupational physicians and competent authorities should promote a better understanding of the non-pharmaceutical interventions able to reduce the risk for human infection, but also urge for an up-to-date assessment of hantavirus seroprevalence in some selected population groups (i.e., agricultural and forestry workers; migrants/refugees, etc.). At the same time, an appropriate inquiry of non-seasonal influenza-like syndromes, as well as acute and chronic renal diseases of unknown etiology in certain occupational groups, may guarantee an early identification of potential outbreaks and spillover, with potential benefits far exceeding occupational settings.

## Figures and Tables

**Table 1:** Summary of the studies included in the meta-analysis. Notes: CS = cross-sectional; CC = case control; AW = agricultural workers; FW = forestry workers; PUUV = Puumala Virus; DOBV = Dobrava–Belgrade Virus; ELISA = enzyme-linked immunosorbent assay; IFA = immunofluorescent assay; WB = Western blotting; EIA = enzyme immune assay; SIA = strip immunoblot assay; WB = Western blotting/immunoblotting; HTNV = Hantaan virus; SEOV = Seoul Virus.

Study	Year	Country	Timeframe	Sample Size	AW (% Positive for Hantaviruses)		FW (% Positive for Hantaviruses)	Methods	Study Design	Commentary
Adesiyun et al. [34]	2011	Trinidad	2010	236	145 (9.4%)		-	ELISA	CS	The study also included 64 abattoir workers (12.4% seropositive status) and 27 office workers (11.1%), with no significant differences between occupational groups. As inclusion/exclusion criteria were not clearly reported, the sample may be limitedly generalizable, even at local level.
Ahlm et al. [50].	1998	Sweden	1990–1991	1573	910 (5.7%)		-	ELISA + IFA	CC	Referents from various rural centers of Sweden (No. 663) were matched among subjects not living or working in agricultural settings. In total, 4.7% of participants had antibodies against PUUV, 3.3% among referents.
Ahlm et al. [30]	1994	Sweden	1990–1991	1583		63 (15.9%)		ELISA + IFA	CS	Authors did not dichotomize AW from FW; no specific analysis of non-occupational exposures was performed. In total, 5.2% of participants had antibodies against hantavirus (4.9% among professionals other than AW/FW). Residents in rural areas had higher risk for seropositive status (OR 1.66, 95% CI 1.02, 2.70).
Akar et al. [35]	2019	Turkey	2016	193	136 (2.2%)		-	ELISA + WB	CS	Sample from 11 forest villages in the high-risk area of Düzce, Turkey, including all subjects aged 18 to 70 years. Occupational status was determined by means of a questionnaire. No clear dichotomization between agricultural and forestry tasks was performed. A seropositive status was confirmed by Western blotting in 6 cases (3.1%; 5 of them PUUV, 1 DOBV).
Amaral et al. [16]	2018	Brazil	2012–2013	240	127 (9.4%)		-	ELISA	CS	Study from high-incidence area in Southeastern Brazil on 240 individuals with no previous history of hantavirus infection. Occupational status was inquired through a questionnaire The study design is unable to clearly dichotomize occupational from residential exposure. The majority of cases was positive towards Andes virus.
Armien et al. [26]	2004	Panama	2001	1346	186 (29.6%)		-	EIA/SIA	CS	Serosurvey among the residents of 4 villages in a high-risk area (No. 1346 participants). Overall seropositivity was 16.9% (14.9% among non-farmers). The study design is unable to clearly dichotomize occupational from residential exposure.
Bergstedt Oscarsson et al. [31]	2016	Sweden	1998	1729	36 (30.6%)		-	ELISA + IFA	CS	Overall prevalence for PUUV seropositivity was 13.4%, 12.8% in occupational groups other than AW, with no significantly increased risk in multivariate analyses.
Elbers et al. [24]	1999	Netherlands	1992	293	191 (1.6%)		-	ELISA	CC	Case-control study performed on a total of 102 veterinarians, with pig farmers as controls. No reference of the general population was made available. No positivity among veterinarians was identified.
Fernandes et al. [17]	2019	Brazil	2010	466	466 (2.6%)		-	ELISA	CS	Serosurvey on individuals from rural settlements. Occupational status was inquired through a questionnaire. The study design is unable to clearly dichotomize occupational from residential exposure. As 21.7% lacked of appropriate sanitation, and around 32.4% collected their garbage instead of burying of burning, authors cannot rule out a non-occupational source of infections.All positive cases were Andesvirus.
Frey-Täger et al. [18]	2003	Chile	2002–2003	846	341 (1.2%)		135 (3.7%)	ELISA	CS	Various areas of the geographic region IX, with a total of 846 participants (overall seropositivity of 0.72%). Occupational status was inquired through a questionnaire. The study design is unable to clearly dichotomize occupational from residential exposure, as 5 of the 6 patients said they had been exposed to rodents or their excreta either at home or work.
Gardner et al. [51]	2005	United States	2000–2001	101	101 (-)		-	SIA	CS	Study from Nebraska. No positive cases were identified. but the study focused on the Sin Nombre Virus, therefore previous infections from other Hantavirus cannot be ruled out.
Gonzales et al. [52]	2000	United States	1999	436	150 (2.0%)		-	ELISA	CS	Study from New Mexico and Western Texas. A total of 3 positive cases were identified, with a further case among 286 non-AW (0.3%). As the study focused on the Sin Nombre virus, previous infections from other Hantavirus cannot be ruled out.
Groen et al. [23]	1995	Netherlands	1972–1994	8892	679 (0.4%)		151 (4.0%)	ELISA	CS	Serosurvey included: 1783 patients with renal diseases from the Netherlands, 2172 individuals with suspected occupational risk for hantavirus infection, and 4474 from control group deprived of suspected risk factors, and 463 military personnel. Overall seroprevalence of 0.9%. Selection criteria are unclear; high-risk for self-selection bias. Study design unable to dichotomize occupational vs. residential exposures.
Hukic et al. [15]	2010	Bosnia and Herzegovina	2009	1331	103 (6.8%)		44 (6.8%)	ELISA	CS	Study from endemic and non-endemic areas in Bosnia and Herzegovina, without previous symptoms of HFRS. Seroprevalence ranged between 0.8% from non-endemic areas to 6.2% in endemic areas. Non-occupationally exposed showed higher occurrence of PUUV compared to DOBV. Higher risk was reported among subjects ex-soldiers.
Jameson et al. [53]	2014	United Kingdom	2008	119	89 (8.2%)		-	ELISA on salivary sample	CS	Residents of Yorkshire and Humber (local transmission of Hantaviruses previously documented). Global seroprevalence of 7.6%. Occupational and non-occupational exposure were difficult to be discerned. Six out of 9 cases were positive for HTNV/SEOV, with 1 PUUV.
Jurke et al. [40]	2015	Germany	2011–2013	722	-		257 (8.9%)	ELISA + immunoblot	CS	Serosurvey among the employees of forestry enterprises from North-Rhein-Westphal region in Western Germany. A total prevalence of 6.0% was identified, being greater in outdoor workers (8.9%), than in 2.7% in office workers.
Kallio-Kokko et al. [54]	2006	Italy	2000–2003	488	-		488 (0.2%)	ELISA + IFA	CS	Study from Trentino Region. Specific tasks were not reported; also inclusion/exclusion criteria were not clearly defined. Of them, only 1 was positive for DOBV.
Lee & Huang [33]	2015	Taiwan	2012–2013	444	149 (2.7%)		-	ELISA	CS	A 1.7% seropositive status was identified among the general population.. The sampling strategy was unclear, with a possible selection bias.
LLedò L et al. [47]	2019	Spain	2016	100	100 (4.0%)		-	IFA	CS	Study from Guadalajara province in Central Spain, including the 95% of the total forestall workforce of the region. No description on actual exposures among reference population was provided
Martens [55]	2000	Germany	1994–1998	2241	17 (-)		984 (0.9%)	ELISA	CS	Varios occupational groups from the German Region of Mecklenburg-Vorpommern (No. 2241).
Mertens et al. [39]	2011	Germany	2008	563	-		563 (9.1%)	ELISA	CS	Study from eastern Germany (Brandenburg). A total of 51 positive cases were identified (22 TULV, 17 DOBV, 3 PUUV, 3 cross-reactive to all sampled viruses, 6 reactive with 2 of sampled viruses). Neither detailed characterization of tasks performed was provided nor information on the housing of participants.
Nimo-Paintsil et al. [20]	2019	Ghana	2010–2011	657	657 (11.7%)		-	ELISA	CS	Study from 13 villages in Ghana (convenience sampling). Occupational status was inquired through a questionnaire.Overall seropositivity for DOBV and PUUV was 12.2% and 11.3%, respectively (no significant differences in various age groups). The study design is unable to clearly dichotomize occupational from residential exposure.
Nuti et al. [21]	1990	Italy	1985–1990	1583	192 (4.7%)		65 (10.8%)	IFA	CS	Serosurvey on healthy residents from central and northern Italy, including subjects at presumptively higher risk because of their occupational exposure. A prevalence of 2.3% was identified, with extensive heterogeneity among participants (i.e., from 0 to 10.7% in foresters from Cadore region). Despite a study design oriented towards occupational exposures, the selection criteria are unclear, with high-risk for self-selection bias.
Nuti et al. [22]	1993	Italy	1987–1991	1146	203 (5.9%)		200 (6.0%)	ELISA	CS	Serosurvey on healthy residents from high-risk areas in Northern Italy (i.e., Cadore, Cortina d’Ampezzo, Pordenone, Eeastern Friuli). A prevalence of 3.9% was identified, 2.3% in residents without. Despite a study design oriented towards occupational exposures, the selection criteria are unclear, with high-risk for self-selection bias
Oldal et al. [41]	2014	Hungary	2011–2013	835	-		835 (3.0%)	ELISA + WB	CS	Serosurvey on FW from 106 sylvicultures in 9 Hungarian counties. Overall prevalence was 3% in males and 2.5% in females. Specific tasks were not reported; also inclusion/exclusion criteria were not clearly defined.
Polat et al. [36]	2020	Turkey	2017	346	278 (0.4%)		-	ELISA + WB	CS	Cross-sectional study on 346 healthy volunteers residents from the villages of Çal (n. 220), Baklan (n. 68), Çivril (n. 54), Bekilli (n. 4) from the province of Denizli, Turkey. Because of the sampling strategy, an oversampling of high-risk subjects was deliberate. Nearly all AW reported either occupational or non-occupational exposures to rodents and their excreta. The study population did not include low-risk or reference subjects.
Romanì Romanì et al. [27]	2020	Peru	2010	250	250 (0.4%)		-	ELISA	CS	Cross-sectional study in a random sample of rice-farmers. Farmers were recruited among participants to an annual event in Peru, San Martin region. High risk of sampling bias.
Ruo et al. [28]	1994	PRC	1987	1811	1811 (12.1%)		-	ELISA	CS	Cross-sectional study among the residents of two villages in the Zhejiang province, mainland China. A total of 1811 subjects participated into the study, with a total seropositivity of 12.1%, the majority of them for Hantaan virus. Prospective assay was also performed, with 2.3% seroconversion rate among seronegative individuals. authors did not specifically defined how many of participants were active farmers, but as the study was focused on “farming communities” all participants were considered participating to farming activities. Behavioral factors increasing the occurrence of interaction with rodents were risk factors for seropositive status.
Sarathkumara et al. [29]	2019	Sri Lanka	2016	666	125 (20.8%)		-	IFA	CS	Cross-sectional study including both subjects affected by renal diseases (n. 154) and community individuals (n. 512 participants) followed by an unmatched case-control comparison among residents in a high-risk area. Seropositive status was identified in 11.9% of community participants and 39.6% of individuals with renal disorders. The study deliberately oversampled seropositive cases as it included patients known renal disorders. In the present estimates, only cases with no known story of renal disorders were therefore included.
Schultze et al. [49]	2007	Switzerland	2002–2003	1693	379 (0.8%)		100 (-)	ELISA + WB	CS	Screening for hantavirus-specific antibodies among occupational high-risk groups including AW, FW, soldiers (n. 103, positive 1.9%), hunters (n. 91, positive 1.1%), and blood donors not exposed from the aforementioned occupational groups (n. 1020, positive 0.5%). No preventive exclusion of high-risk groups among blood bank donors was performed.
Sibold C et al. [56]	1999	Slovakia	1999	2286	-		153 (5.9%)	ELISA	CS	Serosurvey on specimens from 2133 residents from Western and Eastern Slovakia were compared with samples from 153 forestry workers. Serologic prevalence was 0.84% in the general population. As for the study design, no description on actual exposures among reference population was provided.
Stanford et al. [25]	1990	Northern Ireland	1986	407	320 (1.2%)		-	IFA	CS	A total of 407 from 510 farms in Northern-Ireland were sampled and assessed for various pathogens, including Hantavirus, through immunofluorescence. A total of 320 farmers were assessed for hantavirus.
Tagliapietra et al. [43]	2018	Italy	2015	300	-		187 (10.2%)	ELISA + IFA	CC	Serosurvey on 150 people working in the forestry service of the Autonomous Province of Trento, and 150 from donors attending the local blood transfusion clinic. Only FW performing high-risk tasks were included. Risk factors such as gardening, hunting, having a woodshed, wood cutting, dog ownership, and having a rodent companion were collected. In summary, a total of 187 FW were included.
Traavik T et al. [57]	1984	Norway	1981	221	-		106 (7.5%)	IFA	CS	Sera from 106 healthy FW in high-risk areas for nephropatia epidemica in Norway (1981) and from 115 patients with suspected or confirmed nephropatia epidemica. Performed tasks were not reported, and also inclusion criteria were not disclosed.
Van Charante et al. [58]	1994	Netherlands	1989–1990	302			151 (4.0%)	ELISA + IFA	CC	Serosurvey on 151 FW randomly sampled from 750 employees in the maintenance of state-owned woodland, heathland and national parks. Specific tasks were not reported; also inclusion/exclusion criteria were not clearly defined.
Van Cuong et al. [38]	2015	Vietnam	2013–2014	245	181 (3.3%)			IFA	CS	Serosurvey on a cohort of individuals with high levels of occupational and/or residential exposure to rodents and excreta (n. 245). Of them, 181 were AW, 29 were animal health workers, 12 were pig slaughterers, 18 were poultry slaughterers, 5 were rat traders. No reference data from non-exposed subjects were provided.
Vitek CR et al. [48]	1996	United States	1993	140	-		84 (-)	ELISA	CS	Cross-sectional study from 7 National Park sites in the Southwestern United States. Occupational exposures were determined by means of a questionnaire. Of them, 84 were AW with outdoor activities, the remaining were either office supervisors (n. 14), or office workers (n. 42). Non occupational exposures to rodents were reported by 64% of study participants. None of the participants was seropositive to Hantavirus IgG/IgM class antibodies.
Witkowski et al. [19]	2015	Cote d’Ivoire/Democratic Republic of Congo	2006 + 2011	982	356 (3.9%)			ELISA	CS	Cross-sectional study from 16 villages in Cote d’Ivoire (2007) and five villages in DRC (2011), with a total of 982 samples collected. Occupational status was inquired through a questionnaire. An overall seropositivity was estimated in 3.9% for Cote d’Ivoire and 2.4% for DRC. Study design is unable to clearly dichotomize occupational from residential exposure.
Wroblewska-Luczka P et al. [45]	2017	Poland	2011	820	-		594 (1.7%)	ELISA	CS	Cross-sectional study on 820 randomly selected workers from the Polish State Forest Service. Workers were dichotomized in high-risk (outdoor, n. 594) and low-risk (indoor, n. 223) groups by the time spent in office (i.e., 50% cut-off). An overall prevalence of 0.8% was reported among office workers. Very same population of ref. [59]
Wroblewska-Luczka P et al. [59]	2017	Poland	2011	820			594 (3.4%)	ELISA	CS	Cross-sectional study on 820 randomly selected workers from the Polish State Forest Service. Workers were dichotomized in high-risk (outdoor, n. 594) and low-risk (indoor, n. 223) groups by the time spent in office (i.e., 50% cut-off). An overall prevalence of 2.2% was reported among office workers, and 3.4% among high-risk workers for DOBV. Very same population of ref. [45]
Zöller et al. [60]	1995	Germany	1994	14,929			455 (3.7%)	IFA	CS	Cross-sectional study on sera originating from residents of various geographic regions of Southern, Western, and Eastern Germany (n. 13,358), with an overall prevalence of 1.7%. A series of samples were then retrieved from high-risk groups, including occupational ones (i.e., 1284 total samples). Among occupational groups, FW from Baden-Würteemberg (n. 64, 6.4% positive) and from Berling/Brandenburg (n. 392, 3.3% positive) were retrieved. As for the study design, no specific analysis of actual tasks was performed.
Zukiewicz-Sobczak W et al. [45]	2014	Poland	2013	216			148 (6.1%)	ELISA	CS	Cross-sectional study on 216 employees of the Polish State Forest Service. Of them, 148 mainly performed outdoor activities, while 66 were mainly office workers. A total of 9 outdoor workers were positive to Hantaviruses, 5 for DOBV, 3 for PUUV, 1 for both pathogens. No detailed description of outdoor tasks was performed and also the cut-off (i.e., 50% office activity) potentially included low-risk group subjects occupationally exposed.

**Table 2:** Summary of the occupational populations included in the study. Notes: AW = agricultural worker; FW = forestry workers.

	No. of Studies (/42, %)	No. of Sampled Workers (/15,043, %)	No. of Positive Workers (/821, %)
All Studies	42, 100%	15,043, 100%	821, 100%
Studies including AW	20, 47.6%	6672, 44.4%	529, 64.4%
Studies including FW	14, 33.3%	4715, 31.3%	202, 24.6%
Studies including both AW and FW	8, 19.0%	3656, 24.3%	90, 11.0%
Geographic Origin			
Old World, Europe	27, 64.3%	9779, 65.0%	365, 44.5%
Old World, Asia	4, 9.5%	2266, 15.1%	255, 31.1%
Old World, Africa	2, 4.8%	1013, 6.7%	91, 11.1%
New World	9, 21.4%	1985, 13.2%	110, 13.4%
*North America*	*3, 7.1%*	*335, 2.2%*	*3, 0.4%*
*South and Central America*	*6, 14.3%*	*1650, 11.0%*	*107, 13.0%*
Timeframe			
Up to 2000	15, 35.7%	6770, 45.0%	397, 48.4%
2001–2010	12, 28.6%	3655, 24.3%	184, 22.4%
2011–2020	15, 35.7%	4618, 30.7%	240, 29.2%

**Table 3:** Comparison of the seropositive status in agricultural workers and forestry workers by geographic origin. Notes: RR = rate ratio; 95% CI = 95% confidence intervals; OW = Old World (i.e., Eurasia and Africa); NW = New World (i.e., North and Central/South America).

	Agricultural Workers RR (95% CI)	Forestry Workers RR (95% CI)
OW (Europe)	1.000 (REFERENCE)	1.000 (REFERENCE)
OW (Africa)	2.525 (1.945; 3.276)	-
OW (Asia)	3.219 (2.617; 3.959)	-
NW	1.701 (1.321; 2.189)	0.572 (0.238; 1.375)
North America	0.342 (0.110; 1.067)	0.928 (0.389; 2.212)
Central and South America	1.926 (1.494; 2.483)	0.149 (0.009; 2.371)
Up to 2000	1.000 (REFERENCE)	1.000 (REFERENCE)
2001–2010	0.730 (0.597; 0.892)	1.295 (0.936; 1.792)
2011–2020	0.935 (0.768; 1.138)	1.163 (0.880; 1.537)

## Data Availability

The data presented in this study are available on request from the corresponding author.
